# Social Familiarity Governs Prey Patch-Exploitation, - Leaving and Inter-Patch Distribution of the Group-Living Predatory Mite *Phytoseiulus persimilis*

**DOI:** 10.1371/journal.pone.0042889

**Published:** 2012-08-10

**Authors:** Gernot J. Zach, Stefan Peneder, Markus A. Strodl, Peter Schausberger

**Affiliations:** Group of Arthropod Ecology and Behavior, Division of Plant Protection, Department of Crop Sciences, University of Natural Resources and Life Sciences, Vienna, Austria; University of Bristol, United Kingdom

## Abstract

**Background:**

In group-living animals, social interactions and their effects on other life activities such as foraging are commonly determined by discrimination among group members. Accordingly, many group-living species evolved sophisticated social recognition abilities such as the ability to recognize familiar individuals, i.e. individuals encountered before. Social familiarity may affect within-group interactions and between-group movements. In environments with patchily distributed prey, group-living predators must repeatedly decide whether to stay with the group in a given prey patch or to leave and search for new prey patches and groups.

**Methodology/Principal Findings:**

Based on the assumption that in group-living animals social familiarity allows to optimize the performance in other tasks, as for example predicted by limited attention theory, we assessed the influence of social familiarity on prey patch exploitation, patch-leaving, and inter-patch distribution of the group-living, plant-inhabiting predatory mite *Phytoseiulus persimilis*. *P. persimilis* is highly specialized on herbivorous spider mite prey such as the two-spotted spider mite *Tetranychus urticae*, which is patchily distributed on its host plants. We conducted two experiments with (1) groups of juvenile *P. persimilis* under limited food on interconnected detached leaflets, and (2) groups of adult *P. persimilis* females under limited food on whole plants. Familiar individuals of both juvenile and adult predator groups were more exploratory and dispersed earlier from a given spider mite patch, occupied more leaves and depleted prey more quickly than individuals of unfamiliar groups. Moreover, familiar juvenile predators had higher survival chances than unfamiliar juveniles.

**Conclusions/Significance:**

We argue that patch-exploitation and -leaving, and inter-patch dispersion were more favorably coordinated in groups of familiar than unfamiliar predators, alleviating intraspecific competition and improving prey utilization and suppression.

## Introduction

Developing explicit foraging strategies for optimal resource exploitation is a major challenge for every animal. Accordingly, numerous theories strive to predict optimal foraging [1], [2]. For many predators, food is not homogeneously distributed but aggregated in patches, separated by corridors and areas without prey or host items [3], [4]. Consequently, decisions whether to stay or leave a patch with decreasing food and how to distribute among available patches, respectively, are crucial for optimizing resource exploitation and fitness. The optimal strategy is determined by numerous factors such as the current and future quality of present and surrounding patches, the travel time between patches and the risk of dying during travelling [5], [6]. According to Charnov’s [6] marginal value theorem, a forager should leave a food patch when its foraging rate drops below the average intake rate per patch of the entire habitat. The theory predicts the optimal strategy of a single animal without competitors but commonly animals do not forage alone. Moreover, it assumes that the forager has perfect knowledge about the environment, which is never the case in reality [1], [7]. Theories accounting for the presence and influence of other foragers on inter-patch movement and patch occupation are the ideal free and ideal despotic distributions [8], [9]. The ideal free distribution assumes similarity in competitive strengths of foragers, whereas the ideal despotic distribution assumes dissimilarity. Inherently linked to patch exploitation and inter-patch distribution is leaving the natal or original patch and search for new patches to colonize, i.e. dispersal [10].

The ideas of ideal inter-patch distribution linked to optimal patch exploitation and dispersal are especially applicable to group-living animals. Many animals live in groups, often but not exclusively as a result of the patchy distribution of their food [4], [11]. Group-living may yield benefits such as enhanced anti-predator success [12] and/or more efficient foraging [13], but entails also costs such as higher detectability by natural enemies [14], higher risk of disease transmission [15] and increased within-group competition for resources [16]. Group composition and within-group assortment are commonly non-random and may be influenced by group member characteristics such as sex [17], size [18], age [19], social rank [20], genetic relatedness [21] or social familiarity [11]. Thus, one major challenge of group-living animals is to distinguish between group members and adjust patch exploitation, patch-leaving and inter-patch distribution according to group member characteristics. For example, if a patch is inhabited by genetically closely related individuals, dispersal may be a means to reduce inbreeding, avoid kin cannibalism [22] and relax kin competition for shared resources [10], [23]–[25]. The theory of population viscosity states that limited dispersal, i.e. staying disproportionally long in the original patch, increases the relatedness of individuals within this patch. Benefits from altruistic behavior are dispersed to kin within the patch but are limited due to increased local competition [26]–[28]. In hierarchically organized groups, the social rank may determine the onset of dispersal. Weaker or lower-ranking individuals may gain by leaving a group early [29], [30]. However, joining a new group may require the re-establishment of social hierarchy entailing high costs [31]. Another prominent, yet in the context of dispersal and inter-patch distribution rarely addressed feature of group-living animals is social familiarity, independent of genetic relatedness. Social familiarity is based on the ability to learn the phenotypic features of conspecific individuals and allows to discriminate familiar and unfamiliar individuals [32], [33]. Many group-living animals preferentially associate with familiar individuals [11], [34] because it may enhance their performance in foraging [34]–[36], predator vigilance and anti-predator behaviors (Strodl and Schausberger, unpublished data and [37]), or development [38] and reproduction (Strodl and Schausberger, unpublished data). Social familiarity may also reduce agonistic behaviors such as territoriality [39], [40] and intraspecific competition [41]. However, the influence of social familiarity on dispersal, particularly the trade-off between staying and leaving a patch, has been rarely experimentally addressed. The few available studies relate to exploratory behavior, which may be considered a specific form of dispersal if straying from the original site results in permanent leaving. Boldness in exploration has been shown to be influenced by familiarity with environmental features, incl. social familiarity, in domestic chicks, *Gallus gallus domesticus* [42], [43] and guppies, *Poecilia reticulata* [44]. At the cognitive level, heightened boldness in exploration may be explained by attention shifts from otherwise attention demanding inspection of unfamiliar neighbors [45], [46].

Here, we assessed the effects of social familiarity on patch-exploitation, -leaving and inter-patch distribution of the group-living, plant-inhabiting predatory mite *Phytoseiulus persimilis* exploiting two-spotted spider mites, *Tetranychus urticae*. *P. persimilis* is highly specialized on spider mite prey producing dense webbing. Patchy dispersion and group-living of the predators are largely determined by the distribution of its prey [47], but also by mutual attraction [34], [48]. For patch-leaving decisions, *P. persimilis* integrates information from the current patch, such as density of prey and conspecifics [49], and of surrounding patches, such as volatiles indicating the presence of nearby prey [50]. Young gravid females are more dispersive than males and juvenile stages [47] and tend to leave the spider mite patches before local extinction, thereby leaving food for their offspring [51]. As a result of aggregation of the predator eggs within prey patches, after hatching, juvenile predators repeatedly encounter each other and socially familiarize, independently of genetic relatedness [52]. Familiar individuals are treated more favorably in agonistic interactions such as cannibalism [53]–[56]. Moreover, social familiarity may adaptively modulate within-group association, foraging, anti-predator and reproductive behaviors of group-living *P. persimilis* (Strodl and Schausberger, unpublished data and [34]). We hypothesized that social familiarity leads to optimization of local prey patch exploitation, patch-leaving and inter-patch distribution of group-living *P. persimilis*. In the 1^st^ experiment, we examined these behavioral characteristics in juvenile *P. persimilis* under limited prey on detached interconnected leaflets. In the 2^nd^ experiment, we assessed the influence of social familiarity on patch-exploitation, patch-leaving and inter-patch distribution of adult *P. persimilis* females under limited prey conditions on whole plants.

## Materials and Methods

### Origin and Rearing of Experimental Animals

The individuals used for the experiments were offspring from females withdrawn from a laboratory-reared population of *P. persimilis*, which had been founded with individuals field-collected in Greece. The stock population was held on an artificial arena consisting of a plastic tile placed on a water-saturated foam cube (15×15×5 cm) in a plastic box (20×20×5 cm) half-filled with water. The edges of the tile were covered with moist tissue paper. The predatory mites were fed by adding bean leaves (*Phaseolus vulgaris* L.) infested with mixed life-stages of *T. urticae* onto arenas in 2 to 3 d intervals. *T. urticae* was maintained on whole bean plants at room temperature and 16∶8 h L:D photoperiod. Predator rearing units and experimental arenas and cages were stored in environmental chambers at 25±1°C, 60±5% RH and 16∶8 h L:D photoperiod.

### Generating Socially Familiar Predators

Leaf arenas used to obtain *P. persimilis* eggs for the experiments consisted of single leaflets of trifoliate bean leaves placed adaxial surface down on a water-saturated, filter paper-covered foam cube (13×13×4 cm) kept in a plastic box (20×20×5 cm) half-filled with water. Prey was provided by brushing mixed life-stages of *T. urticae* from infested bean leaves onto arenas. To obtain predator eggs of similar age, 30 to 40 gravid *P. persimilis* females were placed onto leaf arenas and allowed to oviposit for 6 h in experiment 1 and 24 h in experiment 2.

In experiment 1, familiarization took place in acrylic cages. Each cage consisted of a circular cavity (15 mm ø) drilled into a 3 mm thick acrylic plate [57]. A fine mesh screen closed the bottom opening of the cavity and a removable microscope slide was used to cover the upper opening of the cavity. The slide and the acrylic plate were held together by a metal clamp. Six *P. persimilis* eggs, randomly withdrawn from the oviposition arenas, were transferred into each cage, together with six eggs of *T. urticae* to avoid cannibalism [58]. After ∼48 h, the predator larvae hatched and after another 14 to 16 h they molted to protonymphs. The predators remained in the familiarization cages until all six individuals had reached the protonymphal stage, which is the first feeding life-stage.

In experiment 2, familiarization took place on leaf arenas constructed similarly as the oviposition arenas. Each arena had an accessible size of 6×6 cm, was infested with mixed life-stages of *T. urticae* and furnished with 20 to 25 *P. persimilis* eggs randomly withdrawn from the oviposition arenas. The predators remained on the familiarization arenas for 8 to 10 days, i.e. until they had reached adulthood and were mated.

### Patch-exploitation and -leaving by Juvenile *P. persimilis* (Experiment 1)

Experiment 1 aimed at examining the influence of social familiarity on patch-exploitation and -leaving of juvenile predatory mites under limited food on detached leaflets. Each experimental unit consisted of four interconnected detached trifoliate bean leaflets, placed on a foam cube (15×15×5 cm) covered with moist filter paper kept in a plastic box (20×20×5 cm) half-filled with water. Leaflets were arranged in a Y-shape, with a leaflet in the center, at the junction of the Y, surrounded by the other three leaflets, at the tips of the arms of the Y, and connected by wax bridges. Before the experiment, one to four adult spider mite females were placed on each arena for 24 h to feed and oviposit. Eggs were reduced to 20 on the central arena and to 6 on each of the three surrounding arenas (in total 38 eggs per experimental unit). The number of prey eggs provided should be sufficient for four juvenile predators to reach adulthood in the whole experimental unit of four leaflets, but be insufficient per each single leaflet [51], and thus stimulate movement among leaflets. After spider mite females had been removed, the wax bridges were constructed by dripping hot wax from a non-fragrant candle in between the leaflets [51], [59]. To start the experiment, groups of four familiar or four unfamiliar protonymphs were released on the central leaf arena of each experimental unit. For each group, familiar protonymphs derived from the same familiarization cage, whereas unfamiliar protonymphs each derived from a different familiarization cage. Only protonymphs of cages where all six individuals were in the protonymphal stage were used for the experiment. After release of the protonymphs, the experimental units were observed after 3.5, 5.5, 8.5, 24.5, 27.5, 29.5, 32.5, 48.5, 51.5, 53.5, 56.5 and 72.5 h. At each observation point and on each leaflet, the number, life-stage and general activity (moving or stationary) of living predators, the number of dead predators and the number of consumed prey eggs, were recorded. Predators were judged dead when their corpses were found on one of the leaflets. The experiment was stopped when all remaining individuals had reached adulthood, or ultimately after 72.5 h. Familiar and unfamiliar groups were replicated 23 times each.

### Patch-exploitation and -leaving by Gravid *P. persimilis* Females (Experiment 2)

Experiment 2 aimed at examining the influence of social familiarity on patch-exploitation and -leaving of gravid *P. persimilis* females under limited food on whole plants. Before the experiment, five *P. vulgaris* plants were grown in 1 liter pots until the first trifoliate leaves were fully developed, which lasted ∼2 to 3 weeks at 25±1°C, 60±5% RH and 16∶8 h L:D photoperiod. All plant parts above the first trifoliate leaves were cut off. Each pot of five plants represented an experimental unit. In order to create homogeneous prey patches for *P. persimilis* within each group of five plants, four gravid spider mite females were placed on the middle leaflet of the trifoliate leaf of each of the five plants to lay eggs, resulting in one prey patch per plant. To confine the spider mites to this leaflet, an adhesive (Raupenleim®, Avenarius Agro) was applied around the petiole of the leaflet. After three days, the spider mite females were counted and removed. To ensure similar numbers of spider mite eggs across pots, only those pots, where at least two spider mite females were still present on each of the five middle leaflets, were used for the experiment. After removal of the spider mite females, hot wax from a non-fragrant candle was dropped on the glue to allow free movement of the predatory mites on the petiole.

The experiment was started by releasing groups of either five familiar or five unfamiliar gravid *P. persimilis* females on the middle leaflet of the trifoliate leaf of one plant of each pot (i.e. five *P. persimilis* per group of five plants). Plant pots were randomly assigned to familiar and unfamiliar predator groups. For each group, familiar females derived from the same familiarization arena, unfamiliar females derived each from a different familiarization arena. After releasing the predators on the plants, the middle leaflets harboring the spider mite patches were examined after 24, 48 and 72 h. At each observation, the number of adult *P. persimilis* females present in each spider mite patch was recorded. At the end of the experiment (after 72 h), the number and life-stages of predatory mite offspring and the number of spider mite eggs and mobile juveniles were counted on each leaflet. Familiar and unfamiliar groups were replicated 31 times each.

### Statistical Analyses

All statistical analyses were performed using SPSS 15.0.1 for Windows (SPSS Inc., Chicago, IL, USA, 2006).

In experiment 1, separate generalized linear models (GLM) were used to analyze the effects of familiarity on the number of predators reaching adulthood (out of initially four; binomial distribution with identity link), the predator developmental time (averaged per experimental unit of four leaves; normal distribution with identity link function), the number of exuviae found on the outer three leaves (binomial distribution with identity link), the number of dead individuals (binomial distribution with logit link function) and the number of females reaching adulthood (out of the number of individuals with sex determined; binomial distribution with logit link function). To assess the effects of familiarity on the number of predators dispersed from the central leaf (i.e. found on the outer three leaves), moving predators (yes/no) and on the number of prey eggs left per unit of four leaves (out of initially 38) over time, generalized estimating equations (GEE; binomial distribution with identity link function, autocorrelation structure between observation points) were used. To analyze the effects of familiarity on the predator dispersion index, defined as the number of leaves occupied by at least one mite per unit of four leaves, GEE (binomial distribution with logit link function; autocorrelation structure between observation points) was used. In GEEs, we selected the most parsimonious model with the lowest QIC value [60].

In experiment 2, two-sided Student’s t-tests were used to compare the number of adult *T. urticae* females on day 0, the number of *T. urticae* eggs and juveniles after 72 h and the number of predator juveniles (eggs, larvae and protonymphs) between plant pots harboring familiar and unfamiliar predators after 72 h. Before t-tests, the numbers of spider mites and predatory mites present on the five different plants of a pot were lumped resulting in one number per pot. Generalized estimating equations (GEE; binomial distribution with logit link function; autocorrelation structure between observation points) were used to assess the effects of familiarity on the number of predatory mite females present and their dispersion index, i.e. the number of leaves out of maximal five occupied by the predators per pot, over time. To analyze the influence of familiarity on dispersion of the adult female and juvenile predatory mites between the origin leaf, i.e. the leaf where the predators were initially released, and the external leaves of each pot over time, GEEs (normal distribution with identity link function; autocorrelation structure between observation points and between origin and external leaves, respectively) were used. To this end, the average number of predators per external leaf was calculated before analysis. In GEEs, we selected the most parsimonious model with the lowest QIC value [60].

## Results

### Patch-exploitation and -leaving by Juvenile Predatory Mites (Experiment 1)

**Figure 1:**
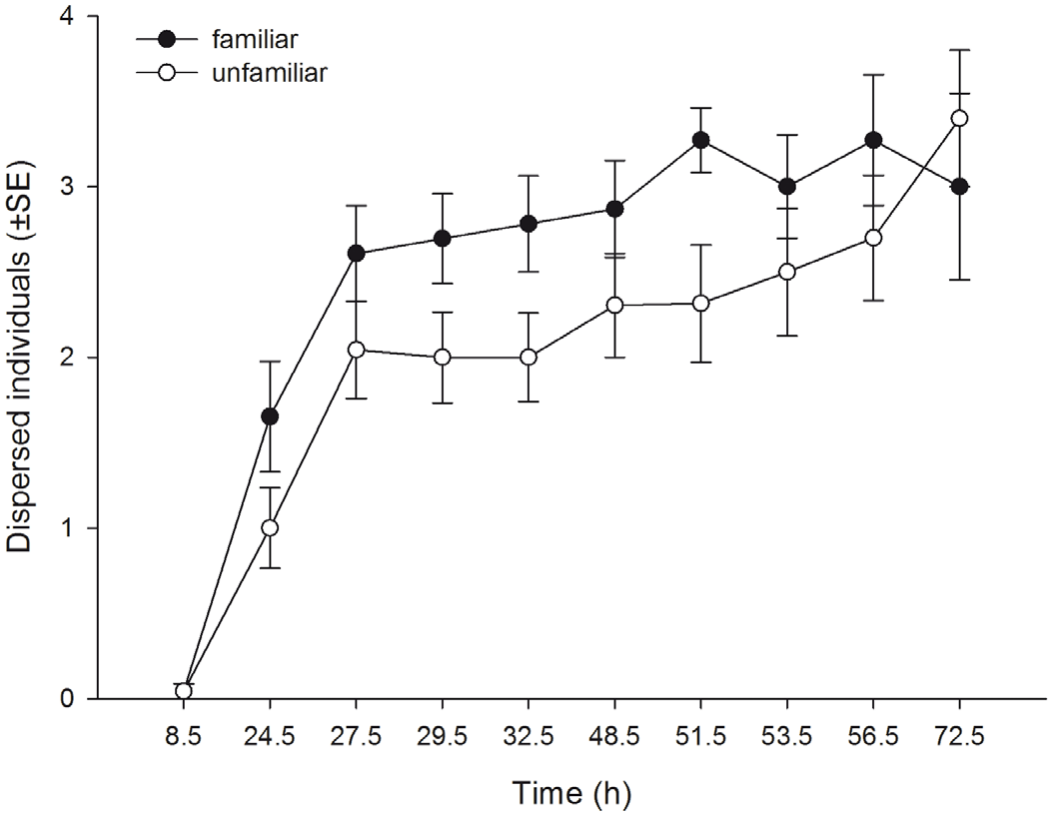
The effects of social familiarity on dispersal of juvenile predatory mites. Number (mean ± SE) of familiar and unfamiliar juvenile *P. persimilis* dispersed from the central leaf (out of 4 individuals initially present) and residing on one of the three outer leaves over time.

**Figure 2:**
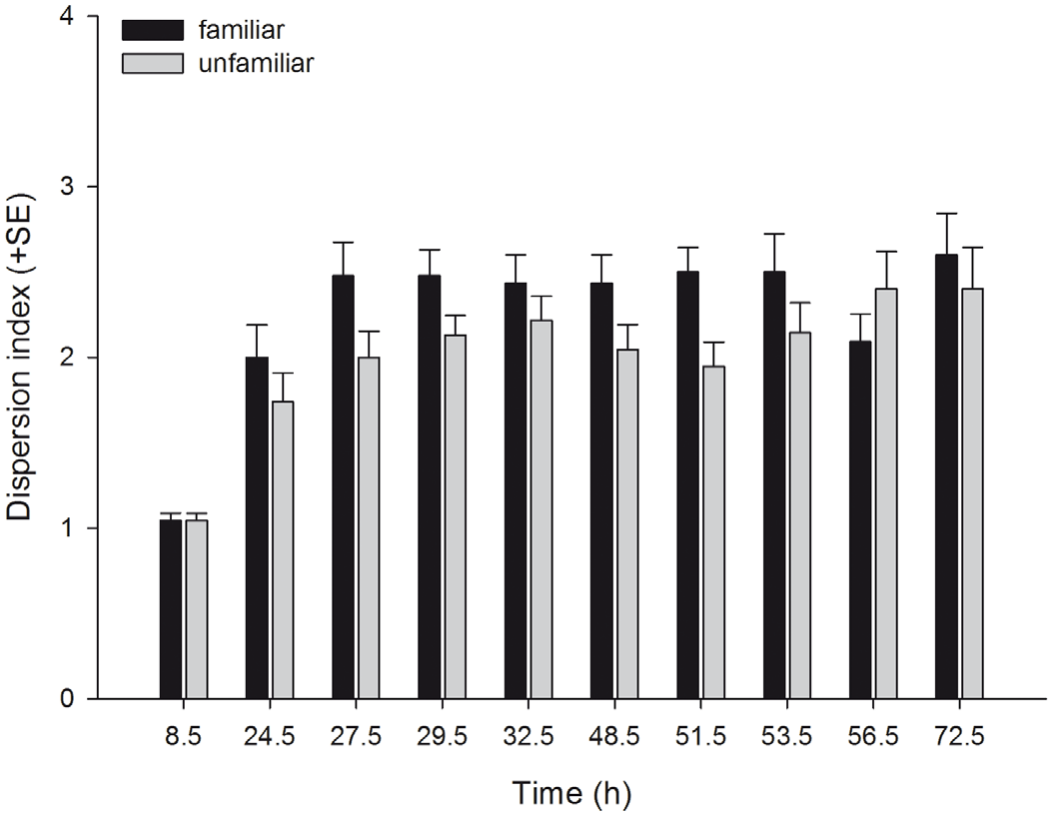
The effects of social familiarity on dispersion of juvenile predatory mites. Dispersion index (mean + SE), i.e. how many leaves out of four leaves per experimental unit were occupied, of juvenile *P. persimilis* held in groups of four familiar or four unfamiliar individuals over time.

**Figure 3:**
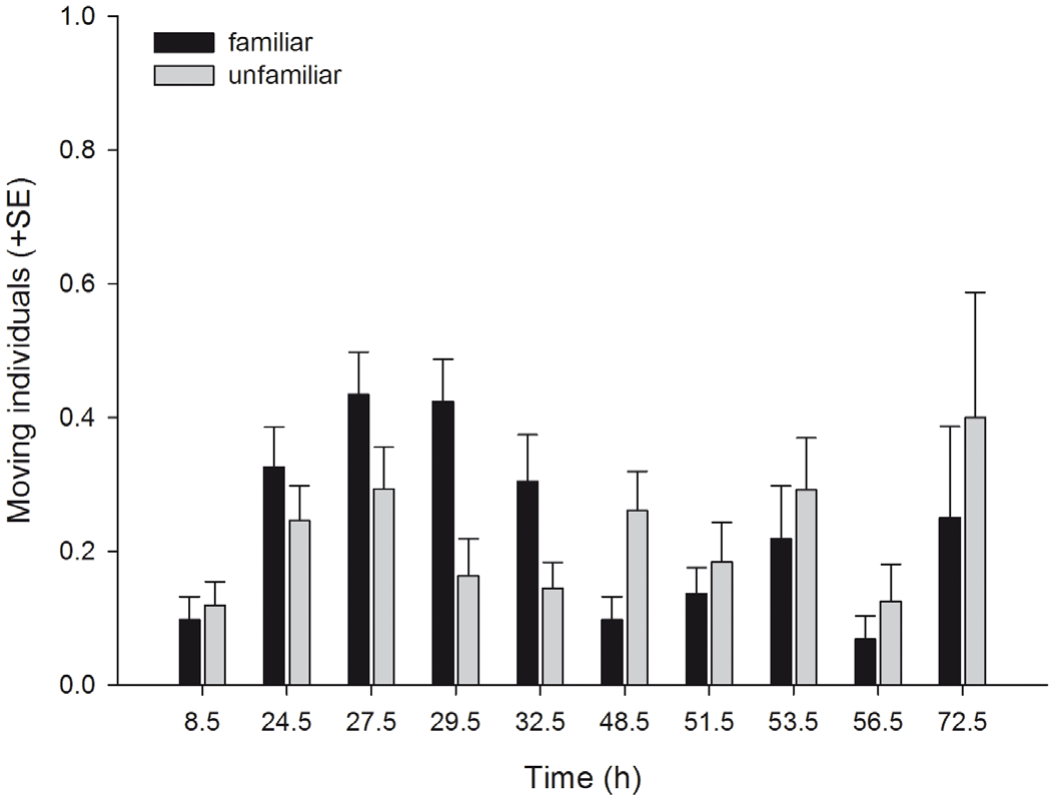
The effects of social familiarity on activity of juvenile predatory mites. Proportion (mean + SE) of juvenile *P. persimilis* moving within groups consisting of four familiar or four unfamiliar individuals over time.

**Figure 4:**
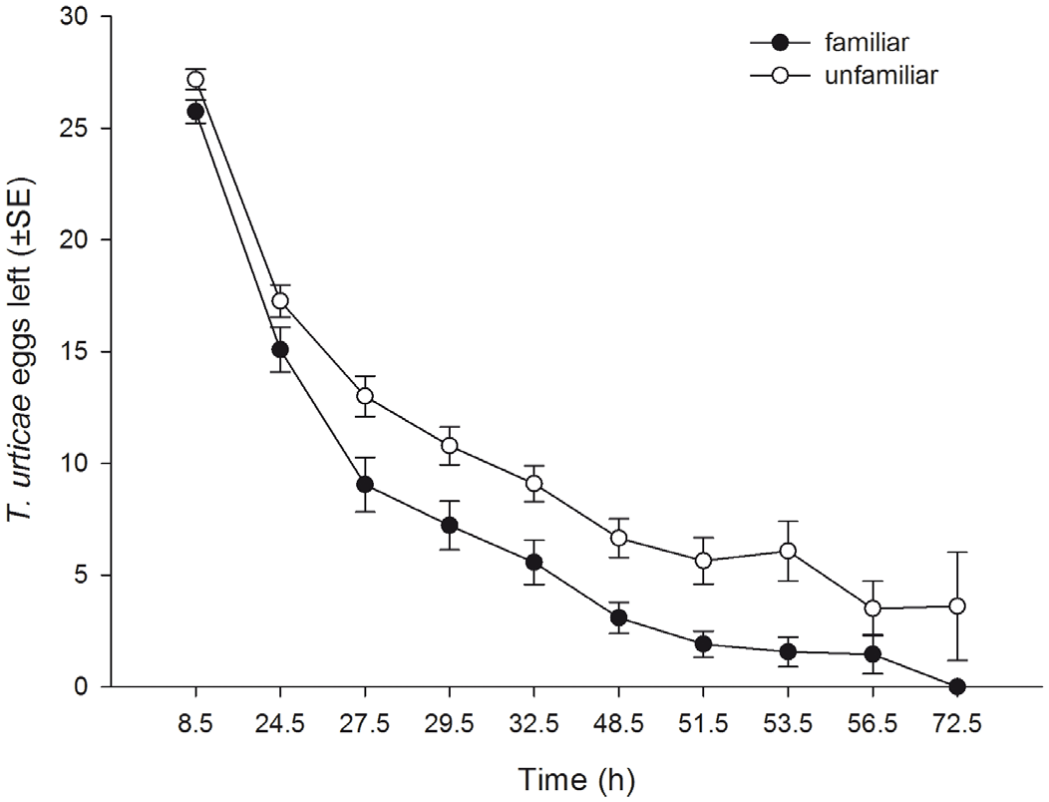
The effects of social familiarity on prey exploitation by juvenile predatory mites. Number (mean ± SE) of *T. urticae* eggs left per experimental unit of four leaves by juvenile *P. persimilis* held in groups of four familiar or four unfamiliar individuals over time.

The number of individuals (mean ± SE per group) reaching adulthood was similar in familiar (3.70±0.16) and unfamiliar (3.52±0.19) predator groups (Wald-χ^2^_1_ = 0.991, P = 0.320). However, the proportion of females among those reaching adulthood (mean ± SE per group) was higher in familiar (0.78±0.06) than unfamiliar (0.57±0.06) groups (Wald-χ^2^_1_ = 4.739, P = 0.029). Familiar and unfamiliar predators needed similarly long to reach adulthood (h, mean ± SE, familiar: 52.19±0.53; unfamiliar: 50.67±1.01; Wald-χ^2^_1_ = 0.272, P = 0.602). Mortality (mean number of dead individuals ± SE per group) was significantly lower in familiar (0.04±0.04) than unfamiliar (0.26±0.11) groups (Wald-χ^2^_1_ = 4.620, P = 0.032). The number of predators dispersed from the central leaf, i.e. those residing on the outer leaves, pooled across time was higher in familiar than unfamiliar groups and dispersal progressed differently over time. Familiar predators started to disperse earlier from the central leaf than unfamiliar predators did (Table 1, Figure 1). As a consequence, also the number of predator exuviae found per outer leaf (mean ± SE) was higher in familiar (0.35±0.03) than unfamiliar (0.24±0.03) groups (Wald-χ^2^_1_ = 5.039, P = 0.025). Familiarity did not affect the dispersion index (i.e. the number of leaves occupied by at least one mite per unit of four leaves) pooled across time, but the dispersion index of familiar and unfamiliar predators progressed differently over time (Table 1, Figure 2). The dispersion index trajectory of familiar predators reached a plateau already after 27.5 h, whereas that of unfamiliar predators increased more gradually. Familiarity had no effect on general activity (moving or stationary) pooled across time, but the activity trajectories of familiar and unfamiliar predators progressed differently over time (Table 1, Figure 3). Familiar predators were more active than unfamiliar predators in the first half of the experiment, whereas the opposite was true in the second half. Familiar predators fed more prey eggs pooled across time and reduced the number of prey eggs more quickly than unfamiliar predators did (Table 1, Figure 4).

**Table 1:** Results of generalized estimating equations (GEE; autocorrelation structure between observation points) for the effects of familiarity and time nested within familiarity on the number of dispersed juvenile predators (i.e. found on the outer three leaflets), their dispersion index (number of leaves occupied), activity (moving yes/no) and number of prey eggs left per experimental unit of four leaflets.

Variable	Source of variation	Wald-χ^2^	df		P
Dispersal	Familiarity	4.228		1	0.040
	Time (familiarity)	1063.341		18	≤0.001
Dispersion	Familiarity	1.688		1	0.194
	Time (familiarity)	365.095		18	≤0.001
Activity	Familiarity	0.020		1	0.888
	Time (familiarity)	111.321		18	≤0.001
Prey eggs left	Familiarity	11.466		1	0.001
	Time (familiarity)	4785.962		18	≤0.001

### Patch-exploitation and -leaving by Gravid Predatory Mite Females (Experiment 2)

**Figure 5:**
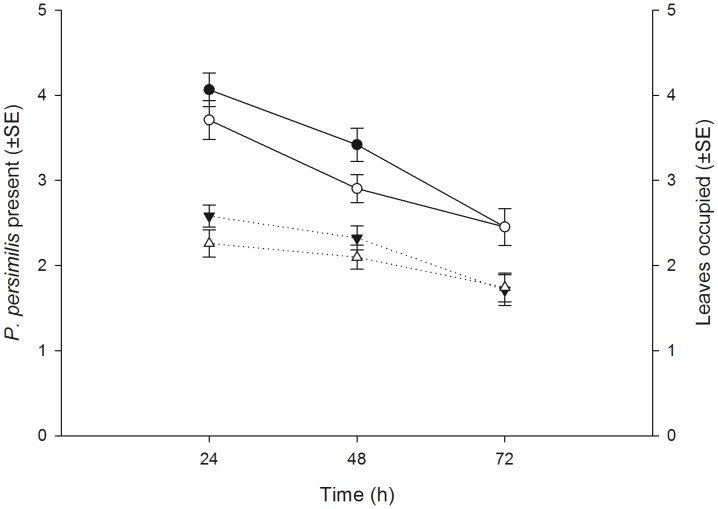
The effects of social familiarity on presence and leaf occupation by predatory mite females. Number (mean ± SE) of familiar (filled symbols) and unfamiliar (open symbols) adult *P. persimilis* females present (solid lines) and the number (mean ± SE) of leaves out of five occupied by them (dotted lines) per experimental unit (group of five plants) over time.

**Figure 6:**
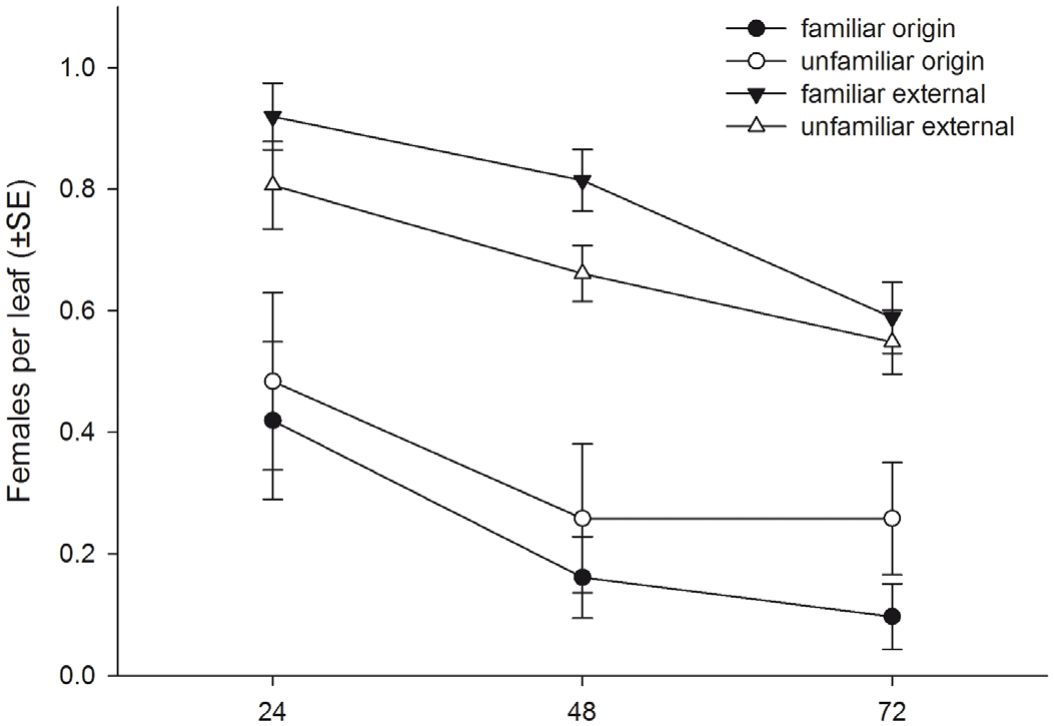
The effects of social familiarity on dispersion of predatory mite females. Dispersion of familiar and unfamiliar adult *P. persimilis* females (mean ± SE per leaf) between the origin leaf, where they were initially released, and the external leaves over time.

**Figure 7:**
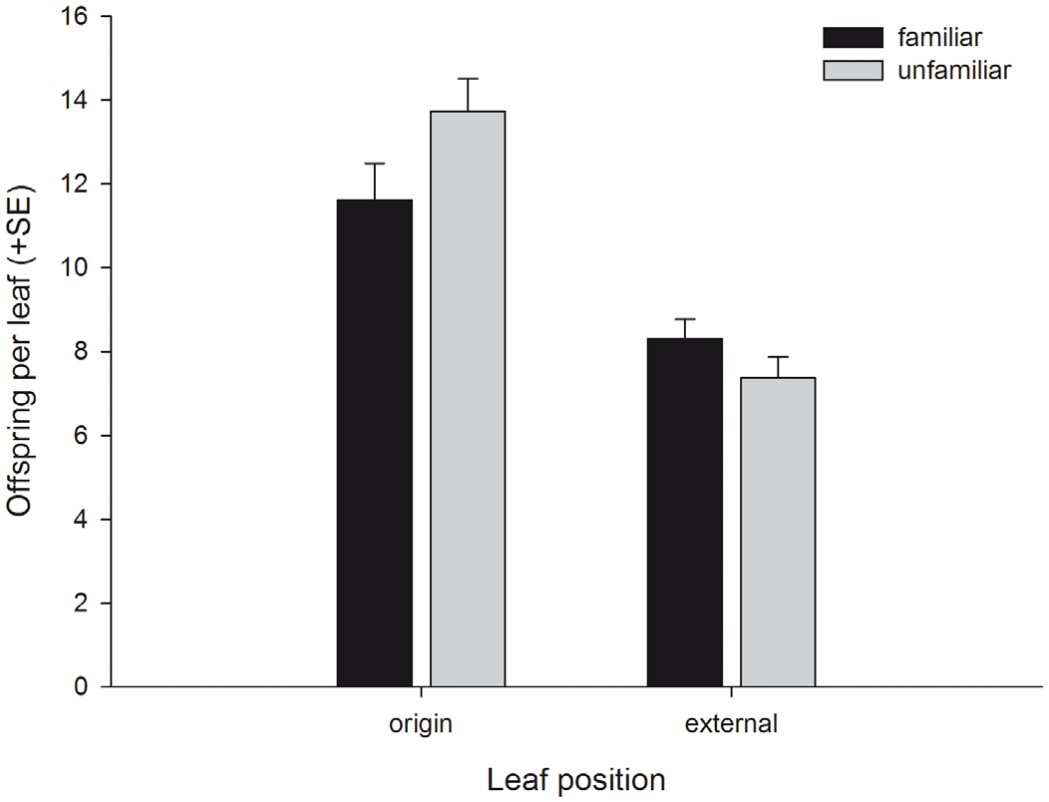
The effects of social familiarity on dispersion of offspring of predatory mite females. Dispersion of offspring (mean + SE per leaf) of familiar and unfamiliar adult *P. persimilis* females between the origin leaf, where the females had been released, and the external leaves at the end of the experiment (after 72 h).

The number of spider mites per pot of five plants (mean ± SE) did not differ between pots assigned to the familiar (18.29±0.36) and unfamiliar (18.68±0.27) predator groups at the beginning of the experiment (Student’s t-test, t_60_ = 0.858, P = 0.394), indicating that initial prey availability was the same for familiar an unfamiliar predator groups. In contrast, at the end of the experiment (after 72 h), the number of juvenile spider mites (eggs, larvae, protonymphs) present per pot (mean ± SE) was significantly lower in familiar (48.37±3.30) than unfamiliar (61.69±3.33) predator groups (t_60_ = 2.842, P = 0.006). The total number of predator offspring (eggs, larvae and protonymphs) produced per pot (mean ± SE) did not differ between familiar (8.97±2.44) and unfamiliar (8.65±2.41) predator groups (t_60_ = −0.534, P = 0.595). Familiarity had no influence on the number of predator females present per plant pot pooled across time but numerical presence of females progressed differently over time, with unfamiliar females leaving the plant groups somewhat earlier than familiar females did (Table 2, Figure 5). Pooled across time, familiar predator females did not occupy more leaves than unfamiliar predator females did. However, the number of leaves occupied by familiar predator females was higher after 24 h and thereafter decreased more steeply over time than the number of leaves occupied by unfamiliar predator females (Table 2, Figure 5). Familiarity had no main effect on dispersion of the predator females between the origin leaf and the external leaves. In both familiar and unfamiliar groups, each external leaf harbored more predator females than the origin leaf. However, the interaction between familiarity and leaf position (origin vs. external) indicates that dispersion of familiar females was more strongly biased towards external leaves than that of unfamiliar females (Table 2, Figure 6). Moreover, while in both groups the number of predator females on the origin leaf, where they were initially released, decreased over time and the number on the external leaves increased over time, familiar predators moved earlier from the origin leaf to the external leaves than unfamiliar predators did. Similar to dispersion of the adult predator females, the dispersion of predator offspring was less strongly biased towards the origin leaf in familiar than unfamiliar groups, indicated by the interaction between familiarity and leaf position (Table 2, Figure 7).

**Table 2:** Results of generalized estimating equations (GEE; autocorrelation structure between observation points) for the effects of familiarity, leaf (origin or external; only for dispersion) and time nested within familiarity (only for females present, leaves occupied and dispersion by females) on the number of *P. persimilis* females present, the number of leaves occupied by *P. persimilis* females and dispersion by *P. persimilis* females and their offspring (after 72 h) between the origin and external leaves.

Variable	Source of variation	Wald-χ^2^	df	P	
Females present	Familiarity	3.233	1		0.072
	Time (familiarity)	56.748	4		≤0.001
Occupied leaves	Familiarity	0.729	1		0.393
	Time (familiarity)	15.169	4		0.004
Dispersion females	Familiarity	0.005	1		0.942
	Familiarity*leaf	72.744	2		≤0.001
	Time (familiarity)	56.709	4		≤0.001
Dispersion offspring	Familiarity	0.764	1		0.382
	Familiarity*leaf	70.224	2		≤0.001

## Discussion

Social familiarity had a decisive impact on spider mite patch exploitation, dispersal and inter-patch distribution of juvenile and adult predatory mites, *P. persimilis*, under limited prey availability. The major trends were similar in experiments with juvenile and gravid female predators, despite using different set-ups and spatial scales – prey patches on interconnected detached leaflets and whole plants. At similar age, familiar juvenile predators dispersed earlier from a limited spider mite patch to adjacent patches than unfamiliar juvenile predators did. Likewise, familiar gravid predator females dispersed earlier from the release prey patch to external patches than unfamiliar females did. Both juvenile and adult familiar predators depleted the spider mite prey more quickly than unfamiliar predators did. In experiment 1, the adaptive significance of social familiarity under the conditions tested, i.e. limited prey distributed among several patches, was apparent in enhanced survival of juveniles and a higher proportion of females reaching adulthood.

### Exploratory Behavior and Dispersal

In experiment 1, all unfamiliar individuals stayed in the release prey patch until molting to the deutonymphal stage, whereas most familiar individuals reached the deutonymphal stage in the patches on the outer leaves, indicating earlier dispersal by familiar juveniles. Experiment 2 revealed a similar tendency of early dispersal in adult predator females. Familiar females left the origin leaf, i.e. the leaf where they were released, earlier than unfamiliar females did. Consequently, the number of predator females decreased on the origin leaf and increased on the external leaves more strongly over time in groups of familiar than unfamiliar females. In *P. persimilis,* two genetically determined types of dispersers have been proposed: leaving before complete prey depletion (“milkers”) and staying until prey is depleted and possibly engaging in cannibalism (“killers”) [61]. Linked to a possible genetic pre-determination, dispersal is extensively modulated by environmental factors [51]. In light of the milker-killer theory, in our experiments familiar females behaved more milker-like than unfamiliar females did, indicated by earlier leaving from the initial prey patch and searching for new prey patches instead of staying and trying to enhance their survival chances and/or advance their developmental stage by cannibalism. The differing patch exploitation and dispersal strategies of familiar and unfamiliar mites are also reflected in the inverse relation of dispersing (familiar more than unfamiliar) and dying (unfamiliar more than familiar) individuals. In experiment 2, familiar females left fewer offspring but more prey per offspring on the origin leaf, which should be advantageous for offspring development on this leaf [51], [62]. An alternative or additional explanation for earlier dispersal by familiar predators in both experiments may be increased boldness. Boldness, the willingness to accept a higher degree of risk in return for potentially higher foraging or reproductive gains, has been found to correlate with familiar environments [43]. Social familiarity increased boldness in guppies [44] and chicks [42], where socially familiar individuals were more exploratory in foraging. In line with these findings, we suggest that in our experiments familiar predators were more prone to explore the surroundings, and thus left the release sites earlier than unfamiliar predators did.

### Inter-patch Dispersion, Prey Exploitation and Competition

In both experiments, social familiarity had a significant influence on spider mite exploitation and prey intake rates. Social familiarity increasing food intake rates has been similarly reported for other animals. For example, red-backed salamanders, *Plethodon cinereus*, had a lower foraging rate in the presence of unfamiliar conspecifics due to spending more time avoiding or interacting with these more aggressive individuals [63]. Likewise, social familiarity led to decreased aggression and higher food intake rates in sea trout, *Salmo trutta* [35]. Interestingly, under ample prey supply, social familiarity had a different influence on *P. persimilis*: juvenile predators living in familiar groups needed less prey during development than those living in unfamiliar groups [34], and females had similar predation rates within familiar and unfamiliar groups (Strodl and Schausberger, unpublished data). It thus seems that the influence of social familiarity on prey intake changes with the possibility to choose between and move to other patches. Proximately, the higher food intake rates of familiar predators are tightly linked to optimized dispersal from the origin patch and inter-patch distribution. Social familiarity led to a better coordination among group members in these inter-related behaviors. With patchily distributed prey, predators are constantly faced with decisions to stay or leave a given patch [64]. Individuals of familiar groups tending to early leave the origin patch with decreasing quality and colonize other resource-rich patches, could thus be interpreted to be more ideally and freely distributed than individuals of unfamiliar groups [8], [65].

### Group-living and Exploitation Competition

In addition to relaxing prey competition due to earlier dispersal and more favorable inter-patch distribution, social familiarity seems to have shifted the type of competition from contest to scramble [66], [67]. In scramble competition, all competitors obtain about the same share of the resources, whereas in contest competition the resources are unequally partitioned among the competitors. For example, Utne-Palm and Hart [41] assessed the effects of familiarity on competitive interactions in sticklebacks. Familiarity decreased the aggressive behaviors leading to lower intraspecific competition and a more balanced food distribution among familiar individuals. We did not particularly assess competition but this may in a similar way apply to our experiments. Unfamiliar predators were less dispersed among patches and exploited them more slowly. Being less dispersed likely intensified exploitation competition and interference in the origin patch and in this way retarded depletion of the prey available on the whole experimental unit by unfamiliar *P. persimilis*. This conclusion is further supported by the higher mortality rates of unfamiliar groups. In contrast, familiar predatory mites dispersed earlier and resource (food and space) exploitation took place in a more balanced way. Thus, on the whole experimental unit, familiar juveniles behaved more scramble competitor-like than unfamiliar juveniles did.

### Group-living, Dispersal and Reciprocity

Under the assumption that premature leaving from a relatively safe site and group initially bears more costs than benefits, such a behavior may be selected for in group-living animals and be adaptively advantageous if the initial costs are later more than compensated for by other group members, i.e. reciprocated [68]. Premature dispersal could then be considered a form of cooperation independent of genetic relatedness. For example, sticklebacks preferred to join individuals that had been cooperative in the past [37]. Individual recognition is a prerequisite for reciprocity between unrelated individuals and this prerequisite is met in *P. persimilis*. Individuals are able to recognize conspecific individuals, which they previously encountered early in life, and treat those familiar individuals, independent of genetic relatedness, more favorably (Strodl and Schausberger, unpublished data and [56]). While the early leavers would pay the costs of premature leaving, the ones that stay would benefit from decreased within-patch competition. The costs can be repaid at a later date when the early leavers and those that stayed in the origin patch meet again in a different patch and the later arrivals take their turns at costly early leaving from this new patch.

### Implications to Biological Control

The knowledge gained in our study could be used to optimize the use of *P. persimilis* as bio-control agents of spider mites or incorporate them in mathematical models to better predict their performance in pest suppression. For example, a common procedure in cultures of the European catfish, *Silurus glanis,* is fish grading, i.e. assorting them in size or quality classes, which usually leads to interactions of individuals that had no previous contact. Grouping familiar fish resulted in reduced conspecific aggression and enhanced energy usage, growth and survival [69]. Similarly, avoiding to release predatory mites from different origins in one and the same crop or trying to package mites with a high likelihood of familiarity in the same bins, could optimize the predator’s performance in spider mite suppression.
